# Factors that influence occupational physicians’ decision to issue an employer warning in Japan

**DOI:** 10.1002/1348-9585.12147

**Published:** 2020-09-02

**Authors:** Naoto Ito, Ayana Ogasawara, Mika Kawasumi, Koji Mori, Tomohisa Nagata, Yoshihisa Fujino

**Affiliations:** ^1^ Occupational Health Training Center University of Occupational and Environmental Health Kitakyushu Fukuoka Japan; ^2^ JFE Steel Corporation East Japan Works Kawasaki Japan; ^3^ Brother Industries Limited, Nagoya Japan; ^4^ Department of Occupational Health Practice and Management Institute of Industrial Ecological Science University of Occupational and Environmental Health Kitakyushu Fukuoka Japan; ^5^ Department of Environmental Epidemiology Institute of Industrial Ecological Science University of Occupational and Environmental Health Kitakyushu Japan

**Keywords:** health risk, Japan, occupational health physician, warning

## Abstract

**Objectives:**

To elucidate the factors that influence occupational physicians’ decision to issue an employer warning.

**Methods:**

The interview was conducted with 10 Japan Society for Occupational Health certified occupational physicians (COPs) and certified senior occupational physicians (CSOPs) to create nine fictive scenarios in which an occupational physician may need to consider issuing a warning. Sixteen CSOPs assessed the seriousness of the problem in each of nine scenarios where they may need to consider issuing an employer warning. Next, using a survey questionnaire, 597 COPs and CSOPs were asked to rate how likely they were to issue a warning in each of the nine scenarios, and answer items on their characteristics and number of previously issued warnings. A multilevel logistic regression analysis nested for various scenarios was used to assess the odds ratio (OR) of being likely to issue a warning.

**Results:**

Valid questionnaires were obtained from 117 participants (19.6%). The ORs and 95% confidence intervals (CIs) were as follows: mean score of seriousness of the problem, 5.90 (4.50‐7.75); years of experience as occupational physician, 1.04 (1.02‐1.06); women, 1.75 (1.20‐2.54); being a part‐time occupational physician without in‐house experience, 2.08 (1.31‐3.29); and having previously issued two or more times warnings, 1.99 (1.29‐3.06), compared with those who had never issued a warning.

**Conclusions:**

Occupational physicians’ likelihood to issue a warning was associated with the seriousness of the problem as assessed in various scenarios, years of experience as occupational physician, gender, employment type, experience as in‐house occupational physician, and number of past warnings.

## 1 INTRODUCTION

The Japanese Industrial Safety and Health Act requires all business operators employing 50 or more workers to appoint an occupational physician. Occupational physicians have the authority to issue warnings (Kankoku; in Japanese)^1^ to the employer and the general manager of occupational health and safety to protect workers’ health. When the employer receives the warnings, the employer shall respect them by the Act.^1^ Warning is the strongest approach that occupational physicians demand on the employer to maintain the health of the workers by law. To respond to challenges such as the decreasing working‐age population associated with the low birth rates and population aging, there have been reforms to the Japanese Labor Standards Act and Industrial Safety and Health Act, collectively enacted under the Work Style Reform Bill.^2^ This bill aimed to reinforce the occupational health functions of occupational physicians to avoid losing workers at high risk of health issues. Since April 2019, occupational physicians have more authority to issue employer warnings. According to the Ordinance on Industrial Safety and Health, when a warning is issued by an occupational physician, the employer's opinion is to be sought in advance regarding the warning, and when the warning is issued, the employer is to document the details of the warning and measures implemented in response to it, whether measures are not taken, and reasons for not implementing any measures. The employer has to report this information to the health committee and conserve the document for 3 years.^3^ Authorizing occupational physicians to issue warnings allows the protection of workers’ health and mandates the employer to report the measures taken to the occupational health committee, which is an important role of occupational physicians, who will make a judgment of their adequacy. Furthermore, the ability to effectively assess health risk, the skills to communicate adequately with employers according to each company and situation, and high ethical standards are important competencies demanded from occupational physicians issuing such warnings.^4^, ^5^, ^6^

However, most occupational physicians who are specialists in occupational health have never explicitly issued a warning to date.^7^ In contrast, 17% of occupational physicians who work as occupational physicians as a second job besides their employment in hospitals or clinics are reported to issue warnings once or more times per year, and 5% of them issue warnings three or more times a year.^8^ This difference seems to be due to the Japanese occupational physician system. In other words, a medical doctor can get an occupational physician license if they take 50 hours of training because there is no examination, but in order to get a license of certified occupational physicians (COPs) from Japan Society for Occupational Health, an occupational physician must pass the examination after having practical experience as an occupational physician and research experience under the guidance of their certified senior occupational physicians (CSOPs). There could be differences in COPs and CSOPs judgments, and it is possible that they may not to exercise this authority appropriately even if they have more authority to issue employer warnings.

Consensus building related to occupational physicians issuing warnings is necessary for this authority to be useful in the effective improvement of occupational health activities. However, current occupational physician activities involve various elements serving employer and workers; thus, it is difficult to build a specific consensus on issuing a warning.

Therefore, it is necessary to identify the factors that influence occupational physicians’ decision to issue employer warnings, and to share the factors in occupational physician training sessions and conferences. These efforts will contribute to narrowing interphysician differences in their decisions and to promote the appropriate exercise of authority to issue warnings, thereby ensuring the health and safety of workers. There is a difference in general risk perception depending on experience,^9^, ^10^ and gender,^11^, ^12^, ^13^, ^14^ however, the factors that influence occupational physicians’ decision to issue an employer warning are not known.

Nine scenarios in which an occupational physician may be required to issue a warning were created in this study to investigate the seriousness of the problem in each scenario, and how occupational physician characteristics and other factors may influence their likelihood of issuing a warning.

## 2 METHODS

### 2.1 Scenarios and seriousness of the problem

A focus group interview was conducted with 10 COPs and CSOPs to create scenarios in which an occupational physician may need to consider issuing a warning. Since the likelihood of issuing a warning may depend on employment type, they were recruited by snowball sampling to take into consideration their employment type; four in‐house occupational physicians, three part‐time occupational physicians with in‐house occupational physician, and three part‐time occupational physicians without in‐house occupational physician.

Nine fictive scenarios were created based on the results of the interview and discussions among three researchers. Subsequently, a preliminary survey was conducted with 13 occupational physicians, including 7 with 1‐3 years of experience and 6 CSOPs in the affiliated institutions of the researchers, to check whether each scenario included the information necessary for considering issuing a warning. Additions and revisions were made to the scenarios accordingly.

The interviews conducted to develop the scenarios revealed the importance that occupational physicians placed on the “seriousness of the problem” when considering whether or not to issue a warning. Therefore, 16 occupational physicians were asked to rate the seriousness of the problem in each of the nine scenarios as a factor that may influence their likelihood of issuing a warning on a 6‐point Likert scale: 1 (extremely low), 2 (low), 3 (somewhat low), 4 (somewhat high), 5 (high), and 6 (extremely high). The seriousness of the problem is determined by the severity of the health risk that workers are exposed to and the seriousness of the company misconduct, and was defined as a factor determining the likelihood of ultimate issuance of a warning. The 16 participants were occupational physicians on the roster of CSOPs recruited by snowball sampling. They had 15 years or more of experience as an occupational physician and their employment fell under three types: seven in‐house occupational physicians, five part‐time occupational physicians with in‐house occupational physician, and four part‐time occupational physicians without in‐house occupational physician.

### 2.2 Questionnaire survey

Since the assessment of the seriousness of the problem may influence physicians’ likelihood of issuing a warning, anonymous survey questionnaires were mailed to 597 occupational physicians on the roster of COPs and CSOPs after excluding the 16 occupational physicians who participated in the abovementioned preliminary survey. Survey items included participant attributes (years of experience as an occupational physician, gender, and employment type), number of past warnings, and likelihood of issuing a warning in the nine scenarios. Since the likelihood of issuing a warning may depend on employment type and vary between in‐house and part‐time occupational physicians, we categorized participants into two broad employment types: (a) in‐house occupational physicians and (b) part‐time occupational physicians who were either affiliated with an educational institution such as a university and had experience as occupational physicians. Part‐time occupational physicians were subcategorized according to whether they had previous experience as an in‐house occupational physician. Participants were asked to rate the likelihood of issuing a warning assuming they were the occupational physician assessing each scenario on a 6‐point Likert scale: 1 (extremely low), 2 (low), 3 (somewhat low), 4 (somewhat high), 5 (high), and 6 (extremely high). A score of 5 (high) or 6 (extremely high) would likely result in an actual warning; thus, a 5‐point score was deemed as the cutoff score for the likelihood of issuing a warning.

### 2.3 Analysis

We estimated the odds ratio (OR) of the likelihood of issuing a warning (ie, having a score of 5 or 6) in a scenario. Since each of the participants indicated their likelihood of issuing a warning in each of the nine scenarios, a multilevel logistic regression was performed nested by scenarios.

The following participant attributes were analyzed as explanatory variables: years of experience as an occupational physician, gender, employment type (in‐house occupational physician, part‐time occupational physician with experience as in‐house occupational physician, and part‐time occupational physician with no experience as in‐house occupational physician), and number of past warnings issued (0, 1, or 2 or more). The mean scores of seriousness of the problem in the scenarios as previously assessed by the 16 CSOPs (other than the participants) were also treated as a unique explanatory variable for the individual scenarios. Stata version 14.0 (Stata Corporation) was used for statistical analyses.

## 3 RESULTS

### 3.1 Development of scenarios

Nine scenarios in which an occupational physician may consider issuing a warning were developed. The types of health risks depicted in the scenarios were anoxia (scenario A), heat stroke (scenario B), pneumoconiosis (scenario C), occupational deafness (scenario D), long work hours (scenarios E and F), lower back pain (scenario G), administrative work at hot room temperature (scenario H), and computer work for extended hours (scenario I) (Supporting Information).

In descending order, the mean scores of seriousness of the problem in the nine scenarios were 5.8 (scenario A), 5.3 (scenario B), 5.1 (scenario C), 4.8 (scenario D), 4.7 (scenario E), 4.5 (scenario F), 4.1 (scenario G), 3.5 (scenario H), and 2.8 (scenario I). The scenarios were named A to I in the same descending order as the mean scores of seriousness of the problem. The 16 CSOP participants had a mean of 20.4 (standard deviation [SD] = 3.7) years of experience as a physician and 17.1 (2.6) years of experience as an occupational physician.

### 3.2 Questionnaire survey

#### 3.2.1 Characteristics of occupational physicians reporting likelihood of issuing a warning

A rate of 19.6% (117/597) of participants provided valid completed questionnaires. The participant characteristics are displayed in Table 1. They had a mean of 23.2 (10.7) years (range: 6‐56 years) of experience as a physician and a mean of 17.7 (8.9) years (range: 4‐40 years) of experience as an occupational physician. A total of 88 were men (75%) and 29 women (25%); 69 (59%) were in‐house occupational physicians, 31 (26%) were part‐time occupational physicians with experience as an in‐house occupational physician, and 17 (15%) were part‐time occupational physicians with no experience as in‐house occupational physician; 89 (76%) had never issued a warning, 9 (8%) had issued it once, 16 (14%) had issued it 2‐4 times, none had issued it 5‐9 times, and 2 (2%) had issued it 10 or more times. Because there were only two occupational physicians who had issued a warning 5 or more times, we classified the number of warnings issued in three categories for analysis: 0, 1, or 2 or more times.

**Table 1:** Characteristics of occupational physicians reporting the likelihood of issuing a warning in scenarios

	Total (n = 117)	%
Years of experience as a physician (mean, SD)	23.2, 10.7	
Years of experience as an occupational physician (mean, SD)	17.7, 8.9	
Gender		
Men	88	75
Women	29	25
Employment type		
In‐house occupational physicians	69	59
Part‐time occupational physician with in‐house occupational physician experience^a^	31	26
Part‐time occupational physician without in‐house occupational physician experience^a^	17	15
Experience of issuing a warning		
0 times	89	76
1 time	9	8
2 or more times	18	15
Missing	1	1

Note

Percentages may not total 100 due to rounding.

^a^ Including those affiliated with an educational institution and experience as occupational physician.

#### 3.2.2 Likelihood of issuing a warning in the scenarios

The scenarios with the lowest and highest mean scores for the likelihood of issuing a warning were scenario I (1.7) and scenario A (4.9), respectively. The rates of participants with scores of likelihood of issuing a warning of 5 (high) or 6 (extremely high) were lowest for scenario I (2%), and highest for scenario A (70%) (Table 2).

**Table 2:** Likelihood of issuing a warning in scenarios

Identifyier	Scenarios	Likelihood of issuing a warning			
	Summary	Mean	Standard deviation	Respondents rating likelihood of issuing a warning as 5 or 6	
				n	%
A	Risk of anoxia with oxygen level of 17.9%	4.9	1.3	81	70
B	Risk of heat stroke at wet‐bulb globe temperature of 37 degrees	4.3	1.3	59	51
C	Conducting the working environment measurement of dust on a factory closure day	4.3	1.4	62	53
D	Sound measurement of the workplace mean 87 dB (A), maximum 95 dB (A)	4.0	1.4	37	32
E	Mild sleep disorder from 60 hours of overtime per month	3.5	1.5	38	33
F	Expected increase in overtime work from the current 60 hours per month	3.2	1.4	25	22
G	Risk of lower back pain among caregivers transporting users alone	3.0	1.3	15	13
H	Office room temperature expected to exceed 27 degrees	2.3	1.1	6	5
I	Computer work for extended hours	1.7	0.9	2	2

Note

A total sample of 117 occupational physicians assessed their likelihood of issuing a warning in each scenario on a 6‐point Likert scale (1 = extremely low, 2 = low, 3 = somewhat low, 4 = somewhat high, 5 = high, and 6 = extremely high).

### 3.3 Factors influencing warning issuance

The ORs and 95% confidence intervals (CIs) for rating the likelihood of issuing a warning as 5 (high) or 6 (extremely high) were as follows for the various explanatory variables: mean score of seriousness of the problem, 5.90 (4.50‐7.75); years of experience as an occupational physician, 1.04 (1.02‐1.06); being a women physician, 1.75 (1.20‐2.54); being a part‐time occupational physician (without in‐house experience), 2.08 (1.31‐3.29), relative to in‐house occupational physicians; and having issued 2 or more warnings, 1.99 (1.29‐3.06), relative to issuing none (Table 3).

**Table 3:** Odds ratio of factors influencing the likelihood of issuing a warning

	Adjusted odds ratio	95% Confidence interval	
Factors related to scenarios			
Mean score of seriousness of the problem^a^	5.90	4.50	7.75
Factors related to occupational physicians			
Years of experience as occupational physician	1.04	1.02	1.06
Gender			
Men	Reference		
Women	1.75	1.20	2.54
Employment type			
In‐house occupational physicians	Reference		
Part‐time occupational physician with experience as an in‐house occupational physician^b^	0.69	0.47	1.01
Part‐time occupational physician with no experience as an in‐house occupational physician^b^	2.08	1.31	3.29
Experience issuing a warning			
0 times	Reference		
1 time	1.11	0.61	2.03
2 or more times	1.99	1.29	3.06

Note

Likelihood of issuing a warning in the nine scenarios A‐I was rated on a 6‐point Likert scale (1 = extremely low, 2 = low, 3 = somewhat low, 4 = somewhat high, 5 = high, and 6 = extremely high). The odds ratio of having a score of 5 or 6 for the likelihood of issuing a warning for each factor was estimated in a multilevel logistic analysis nested by various scenarios.

^a^ "Seriousness of the problem" was defined by the extent of the health impact on workers and company misconduct, and was assessed by 16 senior occupational health physicians on a 6‐point Likert scale (1 = extremely low, 2 = low, 3 = somewhat low, 4 = somewhat high, 5 = high, and 6 = extremely high).

^b^ Including those affiliated with educational institutions and with experience as an occupational physician.

## 4 DISCUSSION

The present study investigated how occupational physicians’ likelihood of issuing warnings may be influenced by the seriousness of the problem, which is determined by the extent of the health risk of workers and degree of company misconduct (scenario factors), as well as other factors such as years of experience as an occupational physician, gender, employment type, and number of previous warnings issued (individual occupational physician factors).

### 4.1 Seriousness of the problem in scenarios

The higher the seriousness of the problem in each scenario, the higher the likelihood of issuing a warning. In the three scenarios A, B, and C, in which the mean score of the likelihood of issuing a warning was over 4, over 50% of participants had scores of 5 or 6. The health risk in these three scenarios was anoxia, heat stroke, and pneumoconiosis. Since all these conditions are potentially fatal, the participants rated them as a very severe problem, which was reflected in their high likelihood of issuing a warning. The health risk was occupational deafness in scenario D, which showed the fourth highest likelihood of issuing a warning; occupational deafness is not fatal, but irreversible. The remaining scenarios (E, F, G, H, and I), which were rated with a lower likelihood of issuing a warning, involved health damages such as lower back pain, which were neither fatal nor irreversible in nature.

Company misconduct was incontestable in scenario C, where working environment measurements were conducted on a closed day. Although the seriousness of the problem for scenario B is higher than scenario C, and the mean score of the likelihood of issuing a warning for scenario B was the same as for scenario C, there were three more physicians in scenario C who rated their likelihood of issuing a warning as five or six than in scenario B. This is suggesting that the number of physicians with scores of five or six increased when there was company misconduct, even if the level of health risk was a little lower.

Therefore, it seems that the seriousness of the problem has two components: the extent of health risk that workers are exposed to and the degree of company misconduct. However, the likelihood of issuing a warning seems to be predominantly affected by the extent of health risk for workers, while company misconduct might increase the likelihood of issuing a warning for some occupational physicians. Risk perception is formed by factors such as fear and unknownness.^15^, ^16^ Risk perception varies among physicians,^17^, ^18^, ^19^ however, the factors were not clear. There are a few reports on the risk perception of occupational physicians in limited situations such as seasonal influenza^20^ and bioterrorism.^21^ This study suggests that occupational physicians consider high risk of workers' health in the order of fatal, irreversible, and reversible illnesses.

### 4.2 Occupational physician factors

#### 4.2.1 Years of experience as occupational physician

The longer participants’ experience as occupational physicians, the more likely they were to issue a warning. In general, those with less experience tend to underestimate risks, and those with more experience tend to avoid risks, whether they are risks in daily life such as driving^9^ or in areas requiring expert knowledge, such as risks associated with medications.^10^ Our findings were consistent with this in that the more years of experience occupational physicians had, the more serious they assessed the health and safety risks to be in the scenarios, and the higher their likelihood of issuing a warning.

It is considered that issuance of a warning by the occupational physician should be the last recourse^10^; indeed, 77% of occupational physicians who participated in the survey had never issued a warning. The more years of experience occupational physicians had, the more confidence they had in their own judgments as occupational physicians. It is possible that they became less hesitant to issue warnings as they learned about the specific processes of issuance from occupational physicians with experience in this matter.

#### 4.2.2 Gender (Reference group: Men)

Women occupational physicians had higher odds of issuing a warning than their women counterparts. Women tend to have higher levels of benevolence and philanthropy than men,^11^ they also tend to be more interdependent,^12^, ^13^ and less likely to engage in risk‐taking behaviors.^14^ Therefore, women occupational physicians may have been more interested in the effects on workers’ health than their men occupational physicians, and thus tried to reduce the health risks, which may explain their higher likelihood of issuing warnings.

#### 4.2.3 Employment type (Reference group: In‐house occupational physicians)

Current or past experience as an in‐house occupational physician influenced physicians’ likelihood of issuing a warning. In‐house occupational physicians have better access to detailed information related to the health and safety of a workplace than part‐time occupational physicians, and as such are able to base their decisions on more varied information. However, the scenarios created for this study were all fictive and short, with a limited word count of 250 English words (400 Japanese characters). Occupational physicians with in‐house occupational physician experience may have been more cautious to say that they would issue a warning based on the scenarios because they may have judged that there was not enough information for a warning, which is considered as the last resort.^22^

Although there were no statistically significant differences in the present analysis, part‐time occupational physicians with no experience as in‐house occupational physicians had a lower likelihood of issuing a warning than in‐house occupational physicians. In‐house occupational physicians often have additional means of fulfilling their roles other than the last resort of issuing a warning; for example, they may be able to check the conditions in the company frequently, and have direct contact with managers and supervisors outside of their normal line of reporting. Part‐time occupational physicians can only check conditions once a month, which may require them to be on the safe side when it comes to assessing health risks.

#### 4.2.4 Experience issuing a warning (Reference group: 0 times)

Occupational physicians with two or more previous warnings had a higher likelihood of issuing a warning. Awareness and decision making related to risks can be influenced by individual personalities^23^ as well as beliefs and worldviews^24^; therefore, it is possible that occupational physicians who have previously issued multiple warnings tend to make higher estimations of workers’ health risks.

### 4.3 Limitations

There are three main limitations to this study. First, only 19.6% of those surveyed returned valid completed questionnaires, which may not reflect the opinions of all COPs and CSOPs. However, the Japan Society for Occupational Health certified associate occupational physicians do not publish the attributes date of COPs and CSOPs which we analyzed as occupational physician factors. The participants had a broad range of years of experience as occupational physicians (4‐40 years), and the sample was representative in terms of participants’ gender and employment type; completed questionnaires were received from occupational physicians with various attributes.

Second, this study surveyed occupational physicians’ likelihood of issuing a warning in fictive scenarios; thus, there could be differences in relation to the actual situations in which occupational physicians would consider issuing a warning. This is because we used nine scenarios and could not exhaustively cover all the situations in which warnings would be issued. Limits to the word count for each scenario also prevented us from including all the information that an occupational physician would collect in their practice, and there may be factors other than those covered in the present survey that may influence whether or not a warning is issued. However, in the preliminary survey, we checked that the scenarios covered the minimum information required to determine whether or not to issue a warning. There were differences between the scenarios in terms of the mean scores of seriousness of the problem, which, as we found in the interview survey, was a factor influencing whether or not to make a warning. Thus, the mean scores for the likelihood of issuing a warning also varied between scenarios.

The third limitation is related to the assessment of seriousness of the problem in each scenario. Since participants were all CSOPs with many being graduates of the same university, it is possible that they have comparatively similar values; thus, our population sample may be biased.

Occupational physicians’ authority to issue a warning is considered as the last resort in advising an employer. Appropriately exercising this authority is an important role demanding occupational physician competency. However, there are almost no occupational physicians who have explicitly issued warnings. Thus, differences between individual occupational physicians in their standards for issuing warnings are expected. The present study succeeded in identifying the factors that influence COPs’ and CSOPs’ decision to issue a warning. We hope that sharing the outcomes of this study in occupational physician training sessions shall contribute to narrowing interphysician differences in their decisions. This study shows that not only seriousness of the problem but also occupational physician attributes influences the issuance of warnings. The experience, gender, and employment type of occupational physicians may also influence physician judgments other than warnings; thus, further research on this topic is warranted. It is necessary to study the risk perception of occupational health specialists in other countries because risk perception changes depending on the race.^25^ This research will be the basic data for that study.

## 5 CONCLUSIONS

Occupational physicians’ likelihood to issue a warning was associated with the seriousness of the problem as assessed in various scenarios, years of experience as occupational physician, gender, employment type, experience as in‐house occupational physician, and number of past warnings. Sharing the factors in occupational physician training sessions and conferences will contribute to narrowing interphysician differences in their decisions.

## CONFLICT OF INTEREST

*Approval of the research protocol:* The present study was approved by the Ethics Committee of Medical Research, University of Occupational and Environmental Health, Japan (September 20, 2018, H30‐110). *Informed Consent:* N/A. The study was completely anonymous, and each of the occupational physicians surveyed voluntarily participated in the study. *Registry and the Registration No. of the study/trial:* N/A. *Animal Studies:* N/A. *Conflict of Interest:* N/A.

## AUTHOR CONTRIBUTIONS

NI, KM, and YF designed the study; NI, AO, MK, and KM conducted a focus group interview; AO and MK created scenarios; NI, TN, KM, and YF analyzed and interpreted the data; NI led the writing.

## Supporting information

Supplementary Material

## ENDNOTE

^1^ Although KANKOKU is translated as “recommendation” in Japanese Law Translation,^1^ “warning” is used in this paper to make it easier to imagine KANKOKU in Japan.
