# Supplementary material for: Factors that influence occupational physicians’ decision to issue an employer warning in Japan
Source: J Occup Health. 2020 Sep 2;62(1):e12147. doi: 10.1002/1348-9585.12147 (PMC7507452; doi:10.1002/1348-9585.12147)
Supplement: Supplementary file 1 — Supplementary Material [file JOH2-62-e12147-s001.docx]

**Supporting Information**

**Scenarios**

**Scenario A**

**Summary of the workplace (factory)**

A company with 800 employees developing electronics. The layout of a basement floor that was previously a storage area is undergoing partial renovation to make space for a new laboratory.

**Case**

A large nitrogen gas cylinder (containing 760 L of liquid nitrogen) was newly installed, and the staff were in the middle of an experiment using nitrogen gas when the occupational physician inspected the basement floor laboratory for the first time. The alarm was ringing as the oxygen meter displayed 17.9%, but they continued with the experiment assuming that a small amount of liquid nitrogen leaking was inevitable while working. The risk assessment before starting the work reported “Risk of residual nitrogen (risk of explosion or anoxia),” but as per discussion with the Health and Safety department, they continued with the tasks under the condition of installing oxygen meters and performing adequate maintenance of nitrogen gas cylinders. They plan to continue with experiments using liquid nitrogen. Several staff assigned to work in the laboratory spend the majority of their working hours in the basement floor laboratory.

The manager and health and safety department manager were instructed of the risk of anoxia in the case of a liquid nitrogen leak accident because the laboratory cannot be immediately ventilated. However, they responded that the measures currently in place were adequate.

**Scenario B**

**Summary of the workplace (factory)**

The factory has 80 employees and is a manufacturer of parts used in a heating apparatus. The factory building is over 70 years old and gets hot in the summer (July-August) and cold in the winter (January-February). Their busy season each year starts in August. Every summer, there is about one employee per week complaining of poor health suspected to be heat stroke. Last year, one employee required emergency transportation to hospital for heat stroke.

The air conditioners, fans, and spot coolers in the factory are maintained regularly, but there are only about half the number of air conditioners required for the factory size.

The wet-bulb globe temperature measurements of the work environment of the worker who required emergency transport last year were 33°C (8:00 AM), 36°C (11:00 AM), 37°C (2:00 PM), 37°C (5:00 PM).

**Case**

The company has been educating the employees at this factory on how to prevent heat stroke, conducting health checks before starting operations, and increasing the frequency of breaks from every 2 hours to every 30 min. However, as of the end of June, there is already about one employee per week who complains of poor health related to heat stroke. The factory manager thought of changing the work uniforms, increasing air conditioning and cooling devices, and installing air showers at the entrance to reinforce heat stroke measures. However, due to budget constraints, the Human Resources and General Affairs division of the company responded that these changes would be postponed for this year.

**Scenario C**

**Summary of the workplace (factory)**

A clay roof tile manufacturing factory with 60 employees has been conducting health checkups for pneumoconiosis and measuring levels of dust in the workplace that is generated in the process of cutting the roof tiles. The occupational physician has been asked to visit the factory after business hours because no one is available to accommodate the visit during work hours. The factory is closed on weekends and holidays.

Working environment measurement results:

1) Measured on April 30^th^ (Monday, a holiday), Control class I*

2) Measured on October 8^th^ (Monday, a holiday), Control class I

Pneumoconiosis health checkup for the five employees who are constantly exposed to dust in their work: All five were classified as Pneumoconiosis Control class I**

**Case**

The factory is not cleaned properly. Dirty dust masks are placed carelessly inside. The company president has been instructed numerous times about the dust measures, but the situation has not improved for over 1 year.

It is indicated on the working environment measurement results that the measurements were all taken on the last day of a 3-day weekend each time. The president answered that this was because “he runs these measurements because there are legal obligations to, but that he does not understand why money and effort should be spent on the working environment measurements, since there have been no claims of lung cancer or other health damage from working in dust, ever.”

*Results of working environment measurements are categorized into three classes: Control class I, indicating that the work environment management is proper; Control class II, indicating that there is room for improvement in working environment control; Control class III, which indicates inadequate working environment control.

**Control class I indicates no findings of pneumoconiosis.

**Scenario D**

**Summary of the workplace (factory)**

A factory with 75 employees owned multiple large press machines that are always operating. Working environment measurements (Sound) were Control class III (Measurement A: 87 dB; measurement B: 95 dB)*. Wearing earplugs is essential in the factory. The factory is currently considering whether or not to conduct a special auditory health examination.

**Case**

The occupational physician had to provide on-site guidance on workers wearing earplugs at several consecutive monthly inspection tours. Despite educating on the risks of occupational deafness multiple times, the factory did not seem to take it very seriously.

Recently, the general health checkups resulted in findings suggesting occupational deafness in 20 employees. Although the factory director was instructed to improve anti-sound measures multiple times, the discussion was at an impasse for 3 months for claims such as “We distribute earplugs. We are considering taking further measures, but this is the busy season, and do not have the time for it now.” The factory director was contacted again, but he gave a similar response.

*Measurement A aims to grasp the average conditions of the workspace, and Measurement B is measured at the spot where worker exposure is estimated to be highest.

**Scenario E**

**Summary of the workplace (office)**

An information technology company with 200 employees, many of whom are system engineers. Chronic overwork has been a problem at the company, where the workload tends to concentrate on specific individuals.

**Case**

A stress check interview was conducted with an employee. This employee has been doing about 60 hours of overtime per month chronically. At times, it exceeds 80 hours, and the employee has felt fatigue for 1 year. He has been asking the manager repeatedly to revise work responsibilities, but without improvements. Depressive mood and mild sleep disorder were noted in the employee; thus, the occupational physician wrote restrict engagement (prohibit overtime) in the stress check interview report submitted to the company. However, although there were some revisions to this employee’s job responsibilities, the employee continues to do around 50 hours of overtime a month for the reason that the business partner highly esteems the abilities of this employee. His sleep disorder has somewhat worsened compared to 2 months earlier.

**Scenario F**

**Summary of the workplace (supermarket)**

Among the 150 employees in a store of a supermarket chain, only 15 are permanent employees. Since the store manager is also an employee, he has no authority to make any decisions regarding the number of employees or business contraction. There is no supervising occupational physician at the company headquarters. Permanent employees are chronically subjected to around 60 hours of overtime per month, but the spirit of “The customer comes first” prevails, and does not help in curbing the practice.

**Case**

The store manager made an inquiry after a permanent employee was hospitalized last month due to recurrent ulcerative colitis.

“I believe that the ulcerative colitis recurrence was due to overwork. Since the last 2-3 years, I have made multiple requests to the director of human resources at headquarters to secure a budget to increase the number of staff, but this is still pending. At that time, I was reprimanded that our store was not performing well, and that we need to increase operational efficiency to boost our sales. We are doing our best to get by, but the load on others has increased to compensate for the missing employee; at this rate, somebody else will collapse at some point. I have been explaining this situation to the human resources director, but he is not understanding. Doctor, I wonder if you could relay that we need some sort of measures for this.”

**Scenario G**

**Summary of the workplace (nursing home)**

A nursing home for the elderly with 65 employees. The majority of caregivers are women in their 40-60s, and many are leaving the job for physical reasons.

**Case**

Some of the caregivers stated that due to a shortage of manpower, it was becoming normal for single caregivers to carry users from the bed to the wheelchair alone. When the occupational physician made a tour of inspection, a caregiver was seen carrying a user by herself. When the workplace manager was questioned about this, the manager answered that although they used assistive devices such as seat-type patient lifts and sliding seats, the number and installation sites were limited. The caregiver leader was instructed to make sure assistive devices be used for transporting patients or patients be transported in teams of two at all times. On several occasions, proposals were made to the clerical supervisor to hire more employees or purchase additional assistive devices, but 6 months later there have been no improvements.

**Scenario H**

**Summary of the workplace (office)**

Administration office of a construction company with 85 employees. The building of the administration office is 40 years old and deteriorating. During the day, the majority of employees are on construction sites and only several male administrative staff are in the administration office.

**Case**

The room temperature inside the administrative office checked by the occupational physician was 27°C despite being June. Although wearing short-sleeved shirts and omitting ties is accepted starting from June as part of the “Cool Biz” campaign, some staff were seen sweating as they worked. The causes for this were believed to be the declining effectiveness of the air conditioners and the office being crowded with electronic devices emitting heat. According to the manager, it was not possible to install a new air conditioning system to save costs. The facility did not get improved, and anti-heat measures still involve simply using fans and rigorous enforcement of rehydration. Every summer, there are days in the months of July and August when the room temperature exceeds 28°C; so far, there are no employees who have had health problems, but the occupational physician believes that further anti-heat measures are ideal in preparation for the approaching summer.

**Scenario I**

**Summary of the workplace (office)**

A system engineering company with 220 employees. It provides system development and operational support services. Employees, the majority of whom are women in their 30-50s, work in the office performing data entry tasks.

**Case**

Interviews with the occupational physician lasted longer than scheduled. When the occupational physician inspected the workplace during what was supposed to be the break time, approximately half of the employees were still working, facing their computers. The amount of data entry work had increased with the recent increase in orders, which was why they were working without taking their full breaks. Although there were no issues in terms of the working environment such as lighting, inadequate break times could result in employees complaining of eyestrain and musculoskeletal disorders. The occupational physician thus instructed the workplace manager to make sure workers took 15-min breaks after 60 consecutive minutes of work. However, the workplace manager answered that “many of the female employees are raising children and cannot do overtime; they take small breaks individually,” and that “they are discussing with the human resources division hiring more workers.” The occupational physician did see that workers were taking small breaks individually during the workplace inspection, since there were no phone inquiries from clients.
